# Pretreatment Lymphocyte Count Predicts Benefit From Concurrent Chemotherapy With Radiotherapy in Oropharyngeal Cancer

**DOI:** 10.1200/JCO.21.01991

**Published:** 2022-04-06

**Authors:** James M. Price, Hitesh B. Mistry, Guy Betts, Eleanor J. Cheadle, Lynne Dixon, Kate Garcez, Tim Illidge, Zsuzsanna Iyizoba-Ebozue, Lip Wai Lee, Andrew McPartlin, Robin J.D. Prestwich, Savvas Papageorgiou, Dylan J. Pritchard, Andrew Sykes, Catharine M. West, David J. Thomson

**Affiliations:** ^1^ Department of Clinical Oncology, The Christie NHS Foundation Trust, Manchester, United Kingdom; ^2^ Division of Cancer Sciences, Faculty of Biology, Medicine and Health, The University of Manchester, Manchester, United Kingdom; ^3^ Department of Histopathology, Manchester University NHS Foundation Trust, Manchester, United Kingdom; ^4^ Department of Clinical Oncology, Leeds Cancer Centre, Leeds, United Kingdom

## Abstract

There is a need to refine the selection of patients with oropharyngeal squamous cell carcinoma (OPSCC) for treatment de-escalation. We investigated whether pretreatment absolute lymphocyte count (ALC) predicted overall survival (OS) benefit from the addition of concurrent chemotherapy to radical radiotherapy.

**PATIENTS AND METHODS:**

This was an observational study of consecutive OPSCCs treated by curative-intent radiotherapy, with or without concurrent chemotherapy (n = 791) with external, independent validation from a separate institution (n = 609). The primary end point was OS at 5 years. Locoregional control (LRC) was assessed using competing risk regression as a secondary end point. Previously determined prognostic factors were used in a multivariable Cox proportional hazards model to assess the prognostic importance of ALC and the interaction between ALC and cisplatin chemotherapy use.

**RESULTS:**

Pretreatment ALC was prognostic for 5-year OS on multivariable analysis (hazard ratio [HR] 0.64; 95% CI, 0.42 to 0.98; *P* = .04). It also predicted benefit from the use of concurrent cisplatin chemotherapy, with a significant interaction between cisplatin chemotherapy and pretreatment ALC (likelihood ratio test, *P* = .04): higher ALC count reduced the 5-year OS benefit compared with radiotherapy alone (HR 2.53; 95% CI, 1.03 to 6.19; *P* = .043). This was likely driven by an effect on LRC up to 5 years (interaction subdistribution HR 2.29; 95% CI, 0.68 to 7.71; *P* = .094). An independent validation cohort replicated the OS (HR 2.53; 95% CI, 0.98 to 6.52; *P* = .055) and LRC findings (interaction subdistribution HR 3.43; 95% CI, 1.23 to 9.52; *P* = .018).

**CONCLUSION:**

For OPSCC, the pretreatment ALC is prognostic for OS and also predicts benefit from the addition of cisplatin chemotherapy to radiotherapy. These findings require prospective evaluation, and could inform the selection of good prognosis patients for a de-escalation trial.

## INTRODUCTION

The worldwide annual incidence of oropharyngeal squamous cell carcinoma (OPSCC) is rising,^1^ largely because of an increased prevalence of high-risk human papillomavirus (HPV) infection. HPV-associated OPSCCs have a better prognosis compared with those caused by smoking and alcohol,^2,3^ with 5-year overall survival (OS) rates of approximately 90% for patients with stage I disease treated with radiotherapy and concurrent high-dose cisplatin chemotherapy, an accepted standard of care.^4^ Unfortunately, around four in five and one in five patients develop severe (ie, Common Terminology Criteria for Adverse Events v5.0 grades 3-5) acute and late toxicities, respectively,^5,6^ encouraging studies to investigate various treatment de-escalation strategies for good prognosis HPV-associated disease.^5-12^ Omitting or substituting concurrent cisplatin chemotherapy is of particular interest, given the potential for severe and potentially life-threatening acute complications and increased risk of late side effects compared with radiotherapy alone.^13,14^ However, this approach proved unsuccessful,^5,6,11,12^ and predictive biomarkers for more personalized treatment de-escalation are needed.

**CONTEXT**

**Key Objective**
Predictive biomarkers are needed to identify patients with oropharyngeal squamous cell carcinoma for treatment de-escalation. This study examined whether there is an interaction between increased pretreatment absolute lymphocyte count (ALC) and reduced survival benefit from having chemotherapy with radiotherapy.
**Knowledge Generated**
Pretreatment ALC was prognostic for overall survival. We found an interaction between ALC and use of concurrent chemotherapy. Patients with a higher ALC had reduced survival benefit from concurrent chemotherapy. An exploratory analysis also showed an interaction with locoregional control.
**Relevance**
Pretreatment ALC should be used in a prospective clinical trial to select patients with oropharyngeal squamous cell carcinoma for treatment de-escalation.

An effective immune response contributes to improved patient outcomes^15^; for example, HPV-associated OPSCCs with low versus high levels of tumor infiltrating lymphocytes (TILs) have a poor prognosis, with survival rates similar to HPV-negative disease.^16^ An impaired immune response reflects systemically as lymphopenia, and a low pretreatment absolute lymphocyte count (ALC) is associated with poor survival outcomes in a number of solid cancers.^17,18^ Pretreatment ALC may be a prognostic biomarker in OPSCC,^19^ but published findings were mixed (Data Supplement, online only).^20-22^ This may reflect small patient numbers, confounders including use of induction chemotherapy, and dichotomizing ALC rather than using as a continuous variable. The neutrophil:lymphocyte ratio (NLR) is an alternative biomarker for systemic immunosuppression, where high absolute neutrophil counts (ANCs) represent inflammation, which is also associated with inferior survival.^23^ There is continued interest in both pretreatment ALC and NLR as biomarkers in OPSCC, but it is not clear whether they are prognostic and/or whether they correlate with TILs.^24,25^

We hypothesized that patients with high pretreatment ALC have a good prognosis and may not derive additional OS benefit from the addition of cisplatin chemotherapy to radiotherapy. Our primary objective was to investigate whether pretreatment ALC predicted benefit from the addition of cisplatin chemotherapy in a large homogeneous OPSCC cohort. Secondary objectives were (1) to compare the performance of pretreatment ALC and NLR as prognostic biomarkers and (2) assess whether pretreatment ALC correlates with pretreatment TILs.

## PATIENTS AND METHODS

### Study Design and Participants

This was an institutionally approved (reference SE18/2316) retrospective observational study with discovery and validation cohorts. Inclusion criteria for both cohorts were newly diagnosed, histologically confirmed OPSCC; treatment with curative-intent radiotherapy (with or without concurrent chemotherapy); and no induction chemotherapy. The discovery cohort were treated at The Christie NHS Foundation Trust (Manchester, United Kingdom) between 2011 and 2018. The validation cohort were treated at The Leeds Teaching Hospitals NHS Trust (Leeds, United Kingdom) between 2013 and 2020. Patients were restaged according to TNMv8 using their TNMv7 stage and tumor p16 status. ALC and ANC data were retrieved for the period from four weeks before commencing radiotherapy to the end of radiotherapy. NLRs were calculated as ANC/ALC. If multiple values were available for pretreatment ALC and NLR, values closest to the radiotherapy start date were chosen.

### Treatment

Treatment details are provided in the Data Supplement. Briefly, all patients had intensity-modulated radiotherapy or volumetric modulated arc therapy and received mainly 60-66 Gy in 30 fractions over 6 weeks (Manchester) or 70 Gy in 35 fractions over 7 weeks (Leeds). Patients at both institutions underwent a response-assessment magnetic resonance imaging scan of the neck or ^18^F-fluorodeoxyglucose positron emission tomography-computed tomography scan at 12-14 weeks after (chemo) radiotherapy followed by clinical examination and nasendoscopy 3-monthly (years 1 and 2), 4-monthly (year 3), 6-monthly (year 4), and yearly (year 5).

### Immunohistochemistry

Where p16 testing was not performed as standard of care, formalin-fixed paraffin-embedded tissue blocks were obtained and retrospectively tested for p16 as described elsewhere.^4^ The formalin-fixed paraffin-embedded blocks received (168/791 from the Manchester cohort) were also analyzed for TILs using multiplex immunohistochemistry. Primary antibodies against pan-cytokeratin (clone AE1/AE3/PCK26) and CD4 (clone SP35) were obtained from Roche Diagnostics, anti-CD68 (1 in 3,000, clone KP1) from Abcam, and anti-CD8 (1 in 300, clone C8/144B) from Agilent DAKO (Santa Clara, CA). Fluorescent immunohistochemistry was conducted using the Ventana Ultra Discovery Autostainer. Multiplex images were acquired using the Olympus VS120-L100-W-12 (Olympus Corporation, Tokyo, Japan). Data analysis was performed using HALO v3.1.1076.429 (Indica Labs, Albuquerque, NM) software. REporting recommendations for tumor MARKer prognostic studies (REMARK) guidelines were followed throughout.^26^

### Statistical Analyses

The primary outcome measure was 5-year OS calculated from start of radiotherapy to death by any cause or censor date. Patients with no event at the time of data collection after a minimum of 2 years follow-up were right-censored. Prognostic factors from a previously derived prognostic model^4^ were collected and multivariable analysis (MVA) was performed using Cox proportional hazards model. Factors included age, Eastern Cooperative Group Performance Status (ECOG PS), Adult Comorbidity Evaluation 27 index score (ACE-27) comorbidity index, smoking history, TNMv8 stage (which incorporates tumor p16 status), and cisplatin use. For all analyses, complete cases were used initially and reported with results subsequently compared with an analysis where missing data were imputed using chained equations.^27^ Because of a high degree of missingness of the hematologic variables, these were not imputed. If differences in inferences were found, this was reported. Competing risk regression, using the method of Fine and Gray, was used to assess locoregional control (LRC) as an alternative end point, with death as a competing event.^28^ The proportional hazards assumption was assessed graphically and if a violation was found, data were transformed and reported. The relationship between ALC or ANC and log hazard was explored for nonlinearity by comparing linear versus nonlinear models (splines) via model likelihoods. Interactions between pretreatment ALC or ANC and cisplatin use were assessed via the likelihood ratio test.

ALC and NLR were evaluated as prognostic biomarkers by comparing model likelihoods of the following models: (1) ANC, (2) ALC, (3) ANC plus ALC, and (4) NLR, with *P* values from the likelihood ratio test and concordance index (c-index) reported. For all Cox proportional hazard models, hazard ratios (HRs) with 95% CIs were reported. All analyses were done using R version 4.0.1.

## RESULTS

### Discovery Cohort

**FIG 1.**
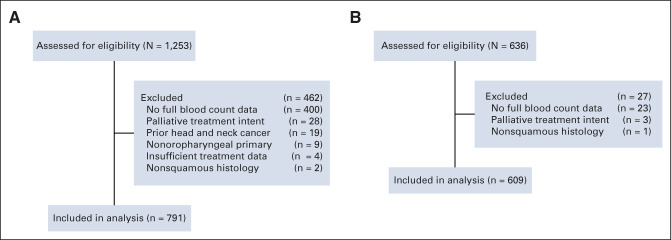
CONSORT diagrams demonstrating how (A) the discovery cohort and (B) the validation cohort were derived.

The discovery cohort comprised 791 patients (Fig 1, Table 1); the median follow-up was 55 months (95% CI, 52 to 59) with 253 events at last follow-up. The distribution of pretreatment ALC did not differ according to clinical factors: age, ECOG PS, ACE-27 comorbidity index score, TNMv8, tumor p16 status, and concurrent systemic therapy type (Data Supplement). Summary statistics for the cisplatin and no-cisplatin groups are in the Data Supplement. The median OS rates at 1, 2, and 5 years were, respectively, 90% (95% CI, 88 to 92), 81% (95% CI, 78 to 83), and 67% (95% CI, 64 to 71). Table 2 shows the results of univariable analysis (UVA) and MVA using Cox proportional hazards model for OS. The relationships between log (hazard) and continuous predictors were linear, showing no natural thresholds for patient stratification. On UVA, many baseline factors were prognostic for 5-year OS. High pretreatment ALC was associated with a good prognosis on MVA (HR 0.64; 95% CI, 0.42 to 0.98; *P* = .04) but not UVA (HR 0.72; 95% CI, 0.47 to 1.09; *P* = .12).

**TABLE 1.**
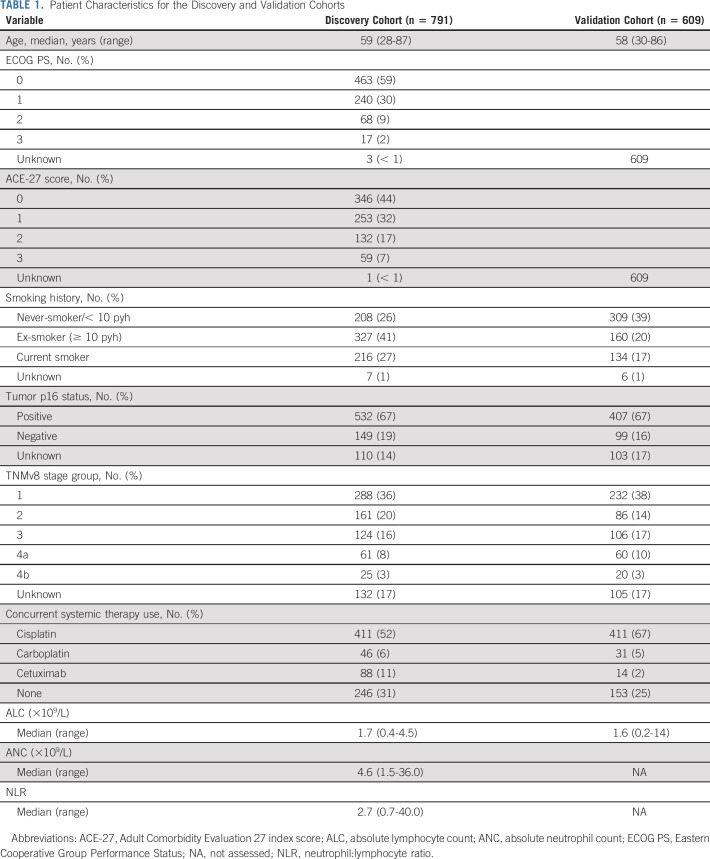
Patient Characteristics for the Discovery and Validation Cohorts

**TABLE 2.**
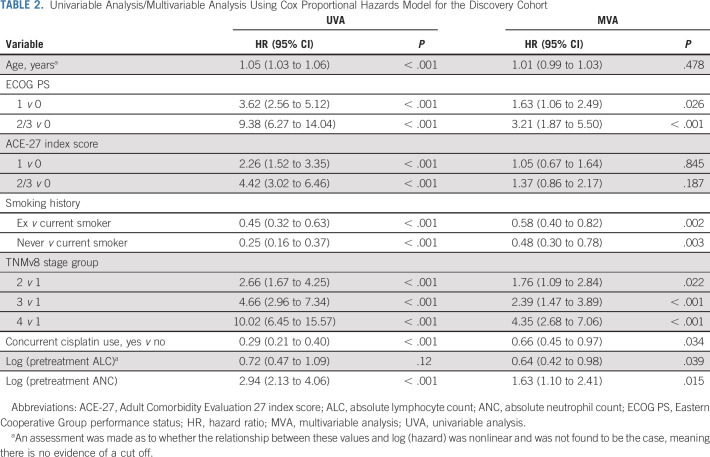
Univariable Analysis/Multivariable Analysis Using Cox Proportional Hazards Model for the Discovery Cohort

**FIG 2.**
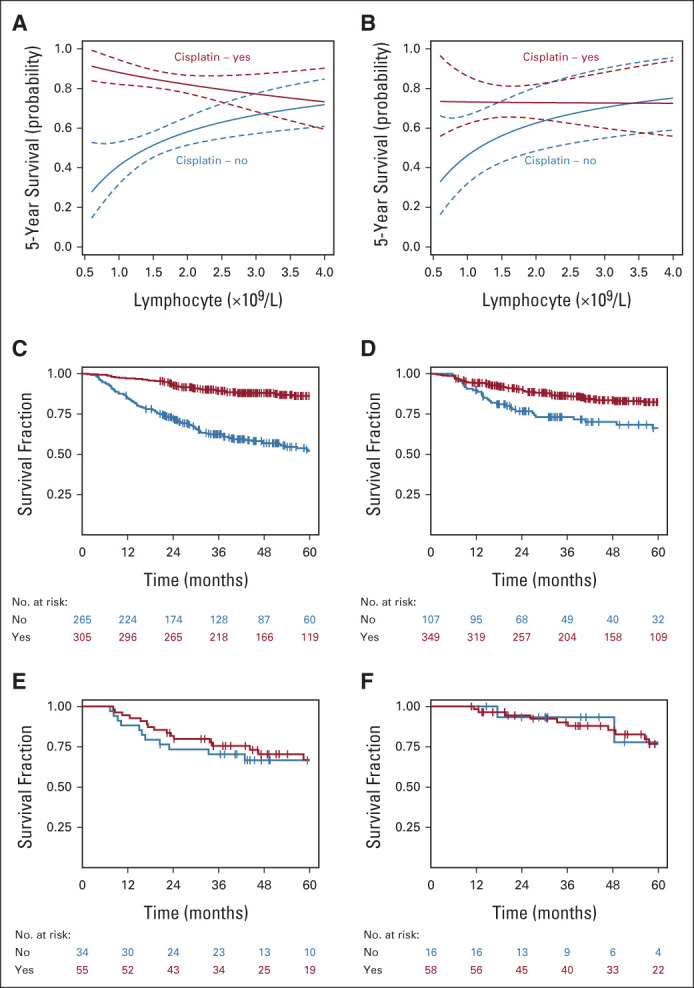
Interaction between pretreatment ALC and benefit from having concurrent cisplatin with radiotherapy. Plots show the 5-year overall survival probability for the pretreatment ALC/cisplatin interaction for the (A) discovery and (B) validation cohorts (calculated using the multivariable model with typical patient values: age = 59 years, performance status = 0, ACE-27 = 0, ex-smoker and TNMv8 stage group 1). Kaplan-Meier curves were generated for patients stratified by ALC ≤ or > 2.4 (where the CIs intersect in A). Patients with low ALC benefit from cisplatin: (C) discovery and (D) validation cohorts (with cisplatin, red line; without cisplatin, blue line). Patients with high ALC did not benefit from cisplatin: (E) discovery and (F) validation cohorts (with cisplatin, red line; without cisplatin, blue line). *x*-axes stop at ALC = 4, as all but three patients in the discovery and validation cohorts had ALC < 4. ALC, absolute lymphocyte count.

**FIG 3.**
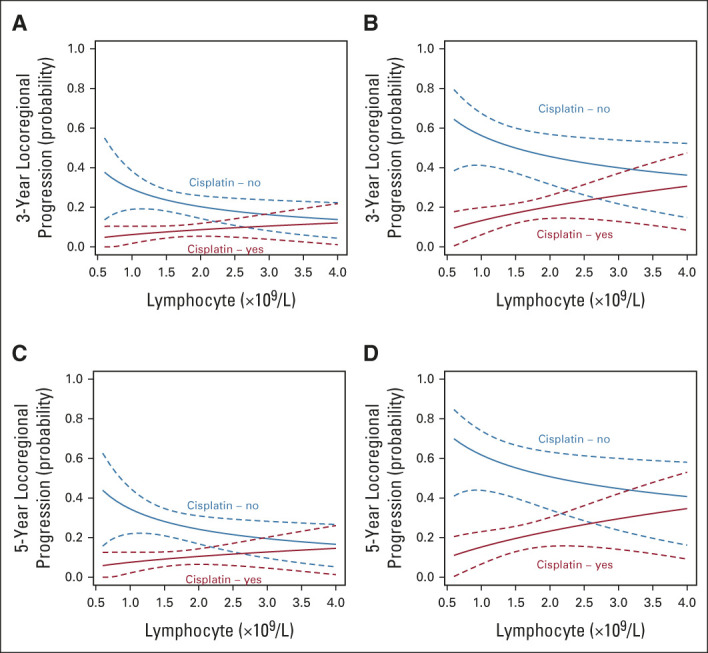
Interaction between pretreatment ALC and benefit in locoregional control from having concurrent cisplatin with radiotherapy. Plots shot the probability of locoregional disease progression at both 3 years for the (A) discovery and (B) validation cohorts and at 5 years for the (C) discovery and (D) validation cohorts. *x*-axes stop at ALC = 4, as all but three patients in the discovery and validation cohorts had ALC < 4. ALC, absolute lymphocyte count.

Pretreatment ALC also predicted benefit from the use of concurrent cisplatin chemotherapy, with a significant interaction between concurrent cisplatin use and pretreatment ALC (likelihood ratio test, *P* = .04; Table 3); that is, the correlation between pretreatment ALC and OS was dependent on whether patients received cisplatin chemotherapy. To further understand this, we plotted the interaction using typical patient values (age = 59 years, PS = 0, ACE-27 = 0, ex-smoker and TNMv8 stage group = 1): Figure 2A shows the lower a patient's pretreatment ALC, the larger the potential benefit of cisplatin. The difference in cisplatin chemotherapy benefit on the basis of this relationship is illustrated (for complete cases) in Kaplan-Meier plots (Figs 2C and 2E) using the intersection of CIs to derive a cutoff value of 2.4. Although TNMv8 stage includes tumor p16 status, separate analyses confirmed no interaction between tumor p16 status and ALC (likelihood ratio test *P* = .73) and that the ALC:cisplatin interaction was also present for the p16 positive-only patient group (Data Supplement). We also assessed the ALC:cisplatin interaction with TNMv8 stage group excluded, and tumor p16 status, tumor (T) stage, and nodal (N) stage included as separate components; the interaction *P* value was .013. The OS:cisplatin interaction was also present at 3 years (Data Supplement) and on competing events regression for LRC, the interaction HR was 2.29 (95% CI, 0.67 to 7.71; *P* = .094; Figs 3A and 3C), suggesting the OS findings are driven by LRC.

**TABLE 3.**
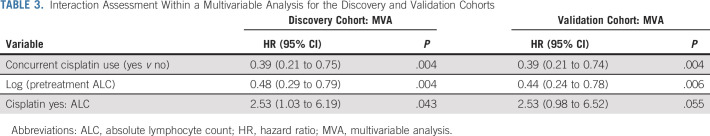
Interaction Assessment Within a Multivariable Analysis for the Discovery and Validation Cohorts

To investigate whether findings for cisplatin chemotherapy might apply to other radiosensitizers, we performed exploratory analyses. Patient numbers were too small to analyze findings for carboplatin and cetuximab alone, but there was a trend for an interaction when assessing radiotherapy plus cisplatin or carboplatin versus radiotherapy alone (interaction HR 2.10; 95% CI, 0.88 to 4.99; *P* = .094). We also assessed radiotherapy plus cisplatin, carboplatin, or cetuximab versus radiotherapy alone and found a further loss of precision of the estimate (HR 1.87; 95% CI, 0.80 to 4.33; *P* = .147). Therefore, our main finding may also apply to concurrent carboplatin chemotherapy as an alternative radiosensitizer, but the role of cetuximab is less clear.

### Validation Cohort

The external validation cohort comprised 609 patients (Fig 1, Table 1). The median follow-up was 51 months (95% CI, 48 to 55) with 162 events at last follow-up. Summary statistics for the cisplatin and no-cisplatin groups are in the Data Supplement. The interaction was assessed adjusting for all prognostic factors available; we could not adjust for ECOG PS and ACE-27 score as these were missing. The interaction was maintained (Table 3, Fig 2), and the HRs from the MVA from both data sets overlap with similar point estimates (Fig 2B). The interaction was also maintained using the alternative end point of 3-year OS (Data Supplement) and for LRC (interaction subdistribution HR: 3.43; 95% CI, 1.23 to 9.52; *P* = .018; Figs 3B and 3D).

### Comparison of ALC With NLR

In the discovery cohort, ANC was a better prognostic indicator than NLR alone with respective likelihood ratio test statistics for ANC, ALC, and NLR of 36.2, 2.40, and 29.9, respectively (Data Supplement). There was no interaction between concurrent cisplatin use and ANC (likelihood ratio test *P* = .390).

### Tissue Analysis

On UVA (Data Supplement), CD8 count was prognostic for OS (HR 0.84; 95% CI, 0.74 to 0.96; *P* = .01). The relationship between CD8 and OS appeared nonlinear; however, there were insufficient events to fully characterize the relationship. The nonlinearity is demonstrated in the Data Supplement, which shows the survival fraction over time of patients stratified into three groups of increasing CD8 counts. Three groups were chosen to ensure there were sufficient events in each arm to quantify survival fractions over time. The relationship between CD8 and OS was maintained after adjusting for age, ECOG PS, smoking history, ALC, concurrent cisplatin use, and TNMv8 stage group (HR 0.83; 95% CI, 0.70 to 0.98; *P* = .028). The lack of change in the HR shows that no other variable contributed to the prognostic information held in CD8 counts. Pretreatment ALC did not correlate with the number of tumor-infiltrating CD4-, CD8-, or CD68-positive cells (Data Supplement).

## DISCUSSION

To the best of our knowledge, this is the first report that pretreatment ALC predicts for a 5-year OS benefit from having concurrent cisplatin chemotherapy with radiotherapy for OPSCC. A low ALC is a poor prognostic factor for patients receiving radiotherapy alone, but is attenuated by cisplatin chemotherapy. Patients with a high ALC do not benefit from having cisplatin chemotherapy: this finding supports our hypothesis and was validated and confirmed in a large independent cohort with virtually identical HRs and CIs.

This study also reports the prognostic significance of pretreatment ALC in OPSCC. Our study addressed the limitations of published studies, where relationships between the ALC and prognosis were mixed (Data Supplement). For example, in a 2015 study of 702 OPSCC cases managed with definitive (chemo)radiotherapy, high pretreatment ALC led to better OS in patients with HPV-positive (n = 510; HR = 0.8; 95% CI, 0.62 to 1.03; *P* = .081) and HPV-negative (n = 192; HR = 0.82; 95% CI, 0.63 to 1.07; *P* = .14) disease; however, precision around the HR was poor.^20^ A recent study by Kreinbrink et al^22^ of 201 OPSCCs treated with primary (75%) or postoperative (25%) radiotherapy found pretreatment ALCs were not associated with OS as either a continuous (HR 0.9; 95% CI, 0.6 to 1.3; *P* = .6) or categorical (HR 0.7; 95% CI, 0.4 to 1.3; *P* = .2) variable. Three other studies assessing the NLR reported prognostic significance, but dichotomized NLR values solely on the sample median.^29-31^ Furthermore, in two studies, event rates were low and CIs were large,^30,31^ and in the other, 45% of patients received induction chemotherapy, potentially confounding results.^29^

Strengths of our study were the larger sample size, including discovery and validation cohorts, excluding cases treated with induction chemotherapy, and analyzing ALC as a continuous variable. The expected imbalance of clinical variables between those who received concurrent cisplatin or radiotherapy alone is a limitation of this work. To minimize the potential for confounding, we included prespecified prognostic factors in the MVA (patient age, ECOG PS, ACE-27 index score, smoking history, and TNMv8 stage).^4^ We also showed that the distribution of ALCs was independent of these clinical factors (Data Supplement). We chose OS as the primary study end point as it is considered robust and unbiased, particularly for retrospective database analyses. Although it is possible that 5-year OS may be affected by noncancer deaths as reported in larynx cancer,^32^ our exploratory competing events regression analyses in both cohorts suggest that our OS findings were driven by locoregional cancer events (Fig 3). Such analyses can be underpowered in retrospective series because of a relatively low local failure event rate (eg, 90 failures in our discovery data set), but the interaction was also replicated in both cohorts for 3-year OS, which compared with 5-year OS may more closely represent cancer-specific survival. However, late toxicity and intercurrent mortality should be assessed formally in future prospective work.

OS appeared lower in the discovery cohort group that did not receive cisplatin chemotherapy; it is possible that these patients were less fit compared with those treated in the validation cohort, although this cannot be quantified as details of performance status (ECOG PS) and comorbidity (ACE-27 index) were not available. There was also a higher proportion of patients in the discovery cohort (2011-2018; 11%) treated with radiotherapy plus cetuximab than in the validation cohort (2013-2020; 2%), probably reflecting the variance in time period for the cohorts and earlier use of cetuximab as a standard of care. This particular variation in practice could mean that patients in the validation cohort that received radiotherapy alone were fitter than those in the discovery cohort. The probability of local failure was lower in the discovery cohort than in the validation cohort (Fig 3). This was due to a lower rate of locoregional failure (Data Supplement) and higher rate of death from any cause (OS events, Data Supplement) in the discovery cohort. Although we cannot fully exclude a difference in treatment approach, importantly, the interactions for both OS and LRC were present in both cohorts.

In our series, ANC better explained the survival data than NLR. ALC was only prognostic after adjusting for numerous clinical variables. Prior studies of NLR did not compare with ANC and/or ALC as independent predictors and hence, we recommend that NLR (or any ratio) is not used without first exploring individual constituents as independent predictors. Most studies transformed ALC and/or NLR from continuous to categorical variables using historical cutoffs or the sample median,^20-22,29-31,33^ increasing the likelihood of a spurious correlation and hence, should be treated with caution.^34^ Reporting an association between survival and ALC/NLR as a categorical but not continuous variable suggests either (1) the relationship is nonlinear or (2) the association was found by chance. We assessed the relationships between log (hazard) and continuous predictors for nonlinearity to determine whether break points occur naturally, but found none; our analyses show that the dichotomy of ALC and NLR is not appropriate.

Although patients with HPV-associated OPSCC experience better survival outcomes compared with HPV-negative disease, standard treatments remain the same for both. The severe additional toxicities and reduced health-related quality of life from the addition of concurrent cisplatin led to de-escalation trials for patients with HPV-associated disease. As only patients with HPV-positive disease are generally considered for de-escalation, we specifically assessed whether the interaction was present for the p16-positive group. The interaction was seen with very similar point estimates, although the number of OS events (n = 100) was less than in the whole cohort (Data Supplement).

The omission of cisplatin chemotherapy or its substitution with cetuximab was not effective in randomized trials, and cisplatin is now accepted to be superior.^5,6,11,12^ Such de-escalation trials defined low-risk disease according to tumor stage (typically T1-3 N0-1 M0) and smoking status (typically never-smokers or < 10 pack-year history) but it remains unclear whether this is the optimal candidate group. Our findings suggest that the pretreatment ALC should be considered for stratification as a surrogate marker of immune status. An alternative de-escalation approach is the substitution of cisplatin chemotherapy for anti–programmed cell death protein-1 or programmed death ligand-1 checkpoint inhibitors.^35^ Combining radiotherapy with immunotherapy may promote immunogenic death by removing checkpoints that inhibit the immune system.^36^

In summary, we demonstrated an association between pretreatment ALC and OS for OPSCC, which correlated with an increased LRC. We also validated the finding that pretreatment levels of CD8 intratumoral T cells are prognostic for OS.^16^ The novel finding of an interaction between the ALC and relative benefit of concurrent cisplatin chemotherapy with radiotherapy suggests, for patients treated with radiotherapy alone, a low ALC is a risk factor for locoregional progression that concurrent cisplatin chemotherapy helps to overcome. It may also infer that patients with a higher ALC do not derive additional benefit from the use of concurrent cisplatin chemotherapy, a hypothesis that requires prospective evaluation.

Although we found no correlation between pretreatment ALC and TILs, the pretreatment ALC may be associated with enhanced dynamic trafficking of T cells from blood into tumor during radiotherapy. In keeping with this, we showed previously that fractionated radiotherapy increases the number of T cells infiltrating a tumor.^37^ However, depletion of ALC is likely to be multifactorial and we cannot exclude depletion because of the large volume elective nodal irradiation used in OPSCC, which is associated with reduced TILs.^38^ As TILs are important for radiotherapy effectiveness,^39^ the proposed association provides a basis for the interaction between the ALC and benefit of concurrent cisplatin chemotherapy with radiotherapy. It is also possible that the immune-modulatory effects of cisplatin chemotherapy help overcome a pre-existing poor immune status. This is supported by the finding that cisplatin leads to cytokine interleukin-1β and chemokine CCL20 signaling and the recruitment of type 3 innate lymphoid cells, which lead to intratumoral infiltration by CD8 lymphocytes.^40^

In conclusion, we show for OPSCC the pretreatment ALC is prognostic and also predicts benefit from the addition of cisplatin chemotherapy to radiotherapy. These findings require prospective evaluation and could inform the selection of good prognosis patients for a de-escalation trial.
